# An exceptionally preserved armored dinosaur reveals the morphology and allometry of osteoderms and their horny epidermal coverings

**DOI:** 10.7717/peerj.4066

**Published:** 2017-11-29

**Authors:** Caleb M. Brown

**Affiliations:** Royal Tyrrell Museum of Palaeontology, Drumheller, AB, Canada

**Keywords:** Dinosauria, Ornithischia, Ankylosauria, Armor, Ornamentation, Allometry, Osteoderm, Keratin, Epidermis, Morphometrics

## Abstract

Although the evolution and function of “exaggerated” bony projections in ornithischian dinosaurs has been subject to significant debate recently, our understanding of the structure and morphology of their epidermal keratinized coverings is greatly limited. The holotype of *Borealopelta*, a new nodosaurid ankylosaur, preserves osteoderms and extensive epidermal structures (dark organic residues), in anatomic position across the entire precaudal length. Contrasting previous specimens, organic epiosteodermal scales, often in the form of horn-like (keratinous) sheaths, cap and exaggerate nearly all osteoderms, allowing for morphometric and allometric analyses of both the bony osteoderms and their horny sheaths. A total of 172 osteoderms were quantified, with osteoderm spine length and height being positively allometric with respect to basal length and width. Despite tight correlations between the different measures amongst all other osteoderms, the large parascapular spines represent consistent outliers. Thickness and relative contribution of the keratinized epiosteodermal scales/sheaths varies greatly by region, ranging from 2% to 6% for posterior thoracics, to ∼25% (1.3×) for the parascapular spines—similar to horn sheaths in some bovid analogues. Relative to the bony cores, the horny portions of the spines are strongly positively allometric (slope = 2.3, CI = 1.8–2.8). Strong allometric scaling, species-specific morphology, and significant keratinous extension of the cervicoscapular spines is consistent with elaboration under socio-sexual selection. This marks the first allometric analysis of ornithischian soft tissues.

## Introduction

Dinosaurs, particularly Ornithischia, bear a startling array of “extreme” and “exaggerated” structures (sensu Gould, 1974; Padian & Horner, 2011; Sampson, 1997) that have sparked interest both in the public and amongst paleontologists. Many of these structures are superficially similar, and perhaps functionally analogous, to those of animals alive today; such as the horns and frill of ceratopsians and those of chameleons and bovids (Farlow & Dodson, 1975; Knell et al., 2013). Still others appear to be without clear parallel in the modern world, such as the plates of *Stegosaurus* (Gilmore, 1914; Marsh, 1896).

Across many ornithischians groups, these exaggerated structures are limited in expression to the skull, and often termed “cranial ornaments”—including the crests of Hadrosauridae, the horns and frills of Ceratopsia, and the domes of Pachycephalosauria. In contrast to this pattern, the diverse and apomorphic osteoderms of Thyreophora are expressed mainly postcranially. Since the first discovery of a thyreophoran dinosaur, the function of the osteoderms has intrigued paleontologists (Mantell, 1833). For the majority of their research history the function of thyreophoran osteoderms has been thought to be as defensive structures, generally, although rarely explicitly stated, in an anti-predation role (Gilmore, 1914; Mallison, 2011; Marsh, 1880, 1896; Moodie, 1910; Sternberg, 1917). As a result, these structures have historically, and ubiquitously, been referred to as “armor” (Brown, 1908; Gilmore, 1914; Lull, 1910; Marsh, 1896). More recently, however, other functions have also been suggested, including thermoregulation (Blows, 2001; Carpenter, 1982; De Buffrénil, Farlow & De Ricqles, 1986; Farlow, Thompson & Rosner, 1976; Hayashi et al., 2010), and antagonistic and socio-sexual display (Arbour, 2009; Arbour & Snively, 2009; Carpenter, 1998; Davitashili, 1961; Gilmore, 1914; Hopson, 1977; Main et al., 2005; Padian & Horner, 2011).

Within Ankylosauria, extensive and *in situ* (anatomically in place) suites of osteoderms have been recovered from several specimens, including the ankylosaurids *Scolosaurus* (“*Euplocephalus*”) (Nopcsa, 1928) and cf. *Pinacosaurus* (Arbour & Currie, 2013b; Carpenter et al., 2011), the nodosaurids *Edmontonia* (Gilmore, 1930) and *Sauropelta* (Ostrom, 1970), and *Kunberrasaurus* (Molnar, 1996, 2001), which has been variously recovered as a basal ankylosaurian or a basal ankylosaurid (Arbour & Currie, 2016; Arbour, Zanno & Gates, 2016; Brown et al., 2017). Although, these osteoderms have been described qualitatively (Carpenter et al., 2011; Gilmore, 1930; Molnar, 2001; Nopcsa, 1928; Penkalski & Blows, 2013), few attempts have concentrated on comprehensively quantifying the size and shape of these structures, and to analyze patterns of variation and allometry.

In life the exaggerated bony structures of ornithischians, whether crest, frill, or horn, or osteodermal plate or spine, would not have been exposed to the external environment, but would have borne epidermal coverings, in the form of epidermal scales—potentially modified into sheath-like structures (De Buffrénil, Farlow & De Ricqles, 1986; Gilmore, 1914). It is this external covering that would have directly interacted with the environment and/or been observed by inter/conspecifics. Due to the limitations of the fossil record, however, documentation and analysis of these exaggerated structures has generally been restricted to the osseous parts of the anatomy, as these are what is generally preserved. Although these epidermal coverings have been occasionally preserved (Arbour et al., 2013; Christiansen & Tschopp, 2010; Gilmore, 1920; Happ, 2010; Hatcher, Marsh & Lull, 1907; Martill, Batten & Loydell, 2000), generally as casts (but see Arbour & Evans, 2017), they are never ubiquitous enough to analyze the patterns of regional variation and scaling. Additionally, recent finds have also illustrated the presence of exaggerated epidermal structures in other ornithischians that do not correlate to obvious underlying bony components (Bell et al., 2014) further highlighting the importance of epidermal contributions to exaggerated structures.

Research on extant animals has illustrated that secondary sexual characteristics, generally associated with intraspecific socio-sexual display, often show strong positive allometric growth/scaling, delayed ontogenetic development, and species specific morphology (Emlen, 2008; Geist, 1966; Lundrigan, 1996). In this context, documenting patterns of relative growth/scaling in dinosaurs exhibiting “exaggerated” structures has been a focus of many paleontologists. This research has shown positive allometric growth/scaling in the crests of Hadrosauridae (Dodson, 1975; Evans, 2010; McGarrity, Campione & Evans, 2013), domes of Pachycephalosauria (Horner & Goodwin, 2009; Schott et al., 2011), and horns and frill of Ceratopsia (Currie et al., 2016; Hone, Wood & Knell, 2016; Lehman, 1990). In contrast to the research on these clades, relative growth/scaling of the exaggerated structures of thyreophoran dinosaurs has been lacking (but see Hayashi, Carpenter & Suzuki, 2009), largely due to a paucity of complete specimens and ontogenetic series.

The recent discovery of *Borealopelta markmitchelli*, a nodosaurid ankylosaur bearing a nearly complete suite of presacral axial osteoderms, and their associated keratinous scales and sheaths (Brown et al., 2017), presents the opportunity to document the morphometrics and allometry of both the bony portions of the osteoderms, and, uniquely, the horny sheaths covering the osteoderms.

## Nomenclature

The nomenclature used to describe the anatomy of postcranial dermal ossifications, and associated soft tissue, in Thyreophora has historically been imprecise and convoluted, with recent attempts to apply more strict definitions to these terms (Arbour et al., 2014). “Osteoderm” is the most ubiquitously applied term, and is defined by Arbour et al. (2014) to indicate a bone developed in the dermis larger than 1 cm (with the term “ossicle” used for elements smaller than 1 cm), with terms such as “plate,” “scute,” “spine,” and “splate” (Blows, 2001) describing different morphotypes of osteoderm. The epidermally derived scale covering the osteoderms has been termed a “keratinous or epidermal scale” (Arbour et al., 2013; Vickaryous & Hall, 2008; Vickaryous & Sire, 2009) or “epiosteodermal scale” (Arbour et al., 2014), the later differentiating this structure from other epidermal scales not associated with underlying osteoderms (“basement scales”). Here, the terms “osteoderm” and “epiosteodermal or epidermal scale” are used, with the knowledge these are roughly equivalent to “osteoscute” and “corneoscute,” respectively, of Francillon‐Vieillot et al. (1990). The term “scute” has been particularly problematic, as this term has been used interchangeably to refer to both the dermal osteoderms (Main et al., 2005; Molnar, 1996), or morphotypes therein (Blows, 2001; Carpenter, 1984; Ford, 2000; Kilbourne & Carpenter, 2005; Kirkland & Carpenter, 1994; Penkalski & Blows, 2013), and the epidermally derived scale (Kardong, 1998; Vickaryous & Hall, 2006). As such this term is avoided.

## Objectives

The first objective of the present study is to quantitatively document the size/shape of individual osteoderms, and link these values to the transverse and longitudinal position of the osteoderm within the complete osteoderm suite.

### Pattern of shape/size change

These data can then be used initially to document patterns of shape/size change within the osteoderm series along transverse or longitudinal axes, or between different body regions. Although previous work has qualitatively discussed changes in osteoderm counts and morphology associated with anatomical regions (or transitions between regions) and along transverse/longitudinal axes in other ankylosaur taxa, these measurements will allow for these patterns to be quantified, and over a large portion of the animal. In addition to raw size/shape data, patterns of variation within individual measures, regions, and rows, can be quantified and compared, allowing for a metric of variation or disparity in osteoderms within and between regions and rows.

### Allometry

In addition to testing how individual aspects of size/shape change within the specimen, bivariate analyses show how different variables are correlated and change with respect to each other. Of particular interest is testing for allometry, the differential scaling of one metric relative to another. This will determine if the osteoderms retain a consistent shape as they increase in size (regionally), or if there are specific shape changes associated with size within the osteoderm series. If specific shape changes are seen, it can be tested if these are consistent in the different osteoderm regions (i.e., are regional differences in shape just brought about simply by size related shape change), or do certain regions show distinct scaling patterns.

It is important to note that the datapoints being used for scaling relationship here are neither specimens of the same species at a single (static/size allometry) or different (ontogentic/growth allometry) developmental points, nor individuals/averages of different, but related, species (evolutionary allometry) (Cock, 1966; Gould, 1966; Klingenberg & Zimmermann, 1992). Rather these data reflect regional allometric scaling within a sample of elements that are, at least at some scale, serially homologous and may best be termed “serial allometry” (Johnson, 1955; LeClair, 1996). To clarify further, rather than the sample being a transect of a population (as in static allometry), an individual’s (or more commonly group’s average) development (as in ontogentic allometry), or the evolution of the lineage (as in evolutionary allometry), this transect represents an anatomic section through a sample of serially homologous elements within a single individual.

Although static, ontogentic, and evolutionary allometry are distinct, they are also necessarily interrelated (Klingenberg & Zimmermann, 1992). It is currently unclear, however, how serial allometry relates to these other forms of allometry. If these structures have a developmental starting point of similar size/shape, the changes in shape related to size across a series of homolog of different sizes at one developmental point, will likely follow a similar trajectory to changes in shape within single elements thought ontogeny. Just how similar these trajectories are is uncertain, but LeClair (1996) present results showing that they may be surprisingly similar. Regardless of the relationship of serial allometry to ontogenetic allometry, it is pertinent to the patterns of relative scaling responsible for creating the varied osteoderm morphologies within the animal (i.e., are the different shapes of the larger osteoderms the result of consistent scaling of the smaller osteoderms, or do these show distinct allometric trends).

Within the context of allometric scaling, isometry (slope = 1) is the null hypothesis, with slopes statistically different from 1 being the result of differential scaling, either positive or negative allometry, relative to another metric.

### Morphospace

Although several ankylosaur species are represented by anatomically in place (though incomplete) osteoderm suites, the armor of a large proportion of known taxa is represented only by samples that are either isolated or displaced both relative to each other and the rest of the skeleton. This lack of data regarding the original position of osteoderms limits the taxonomic and phylogenetic utility of the osteoderms in these taxa.

The preservation of a large number of in place osteoderms in this exceptionally preserved nodosaur allows for testing how well multivariate shape data can predict known anatomic regions and positions of osteoderms. Specifically, if osteoderms pertaining to specific regions and locations are demonstrably segregated in multivariate morphospace (or resulting cluster analyses), or in their categorical assignment, this may provide a broad indication of the level of confidence (or scale) in reconstructing the disassociated osteoderm suites in other taxa (i.e., comparing in place and dissociated specimens within the same taxon).

## Materials and Methods

### Osteoderm nomenclatural scheme

**Figure 1:**
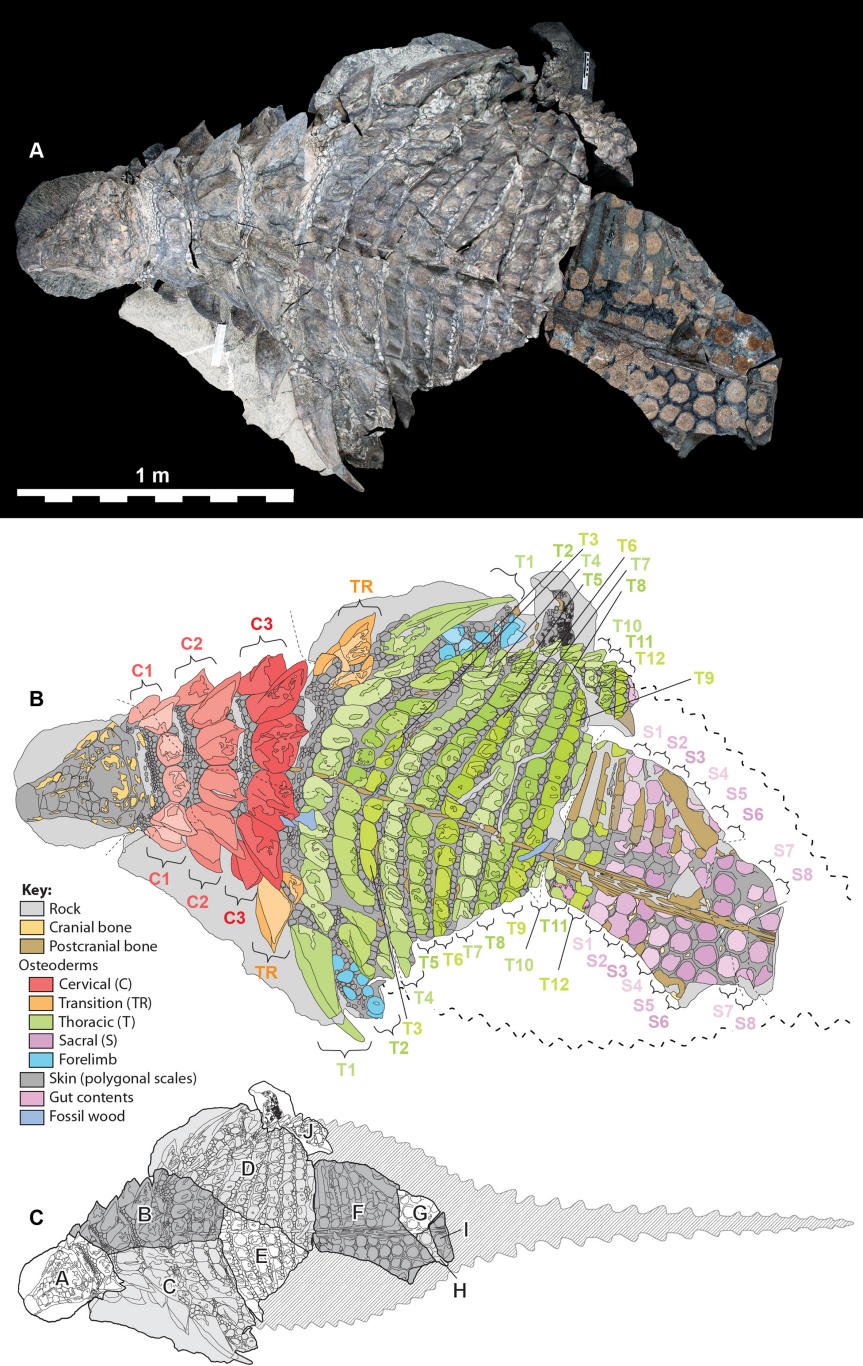
Dorsal view of TMP 2011.033.0001, showing both photocomposite and schematic line drawing. (A) Photocomposite dorsal view of TMP 2011.033.0001. (B) Schematic line drawing of (A) showing osteoderm regions by color. (C) Inset showing constituent blocks of TMP 2011.033.0001, and their relative position within a body outline in dorsal view. Photocomposite (A), created using separate, orthogonal images of blocks A–C, D, E, F–I, and J and combined digitally to reduce parallax. Blocks F, G, H, and I represent reflected counterpart.

**Figure 2:**
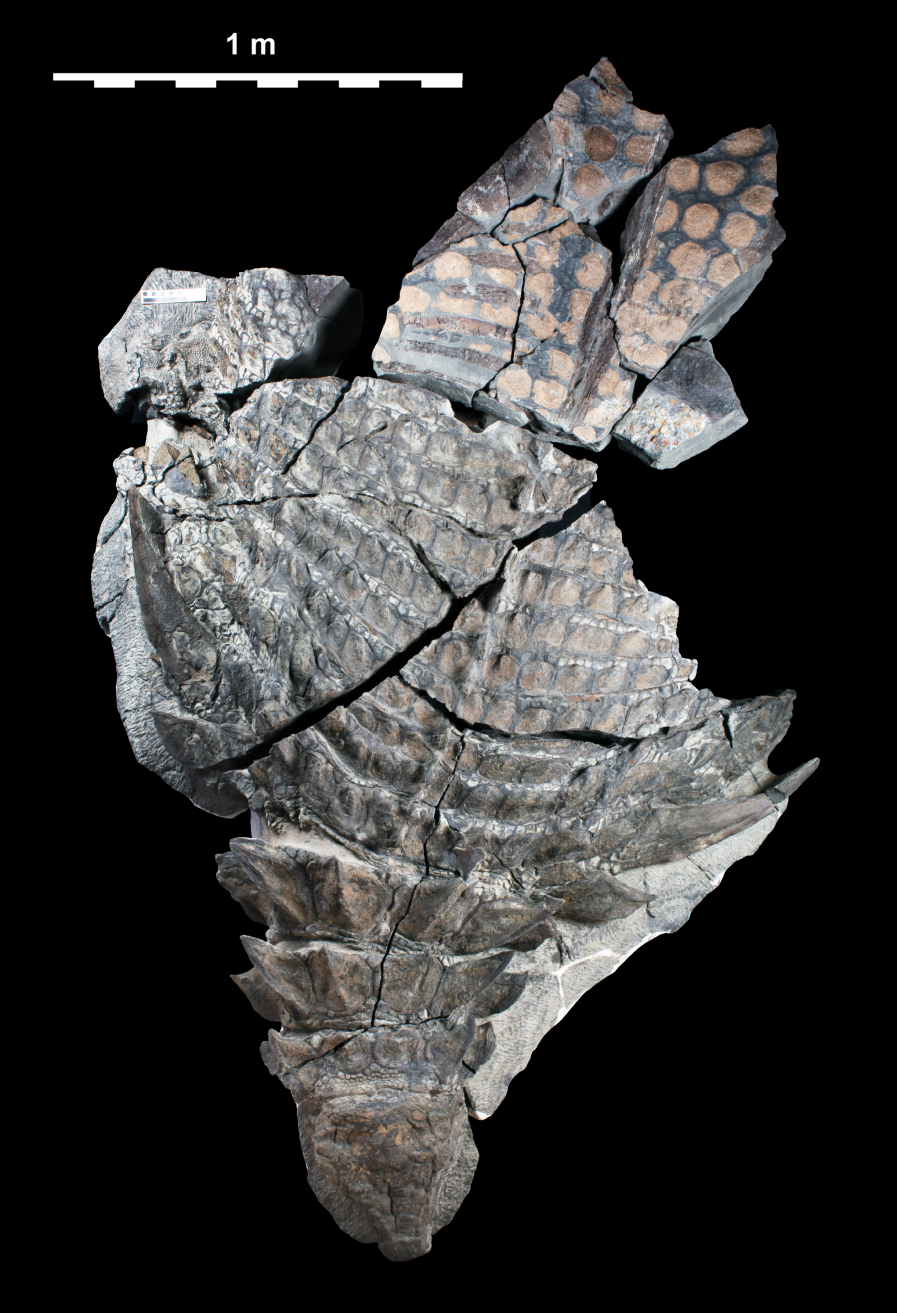
Single dorsal photograph of TMP 2011.033.0001. Sacral region represents original part—reflected counterpart shown in Fig. 4. Scale equals 1 m.

**Figure 3:**
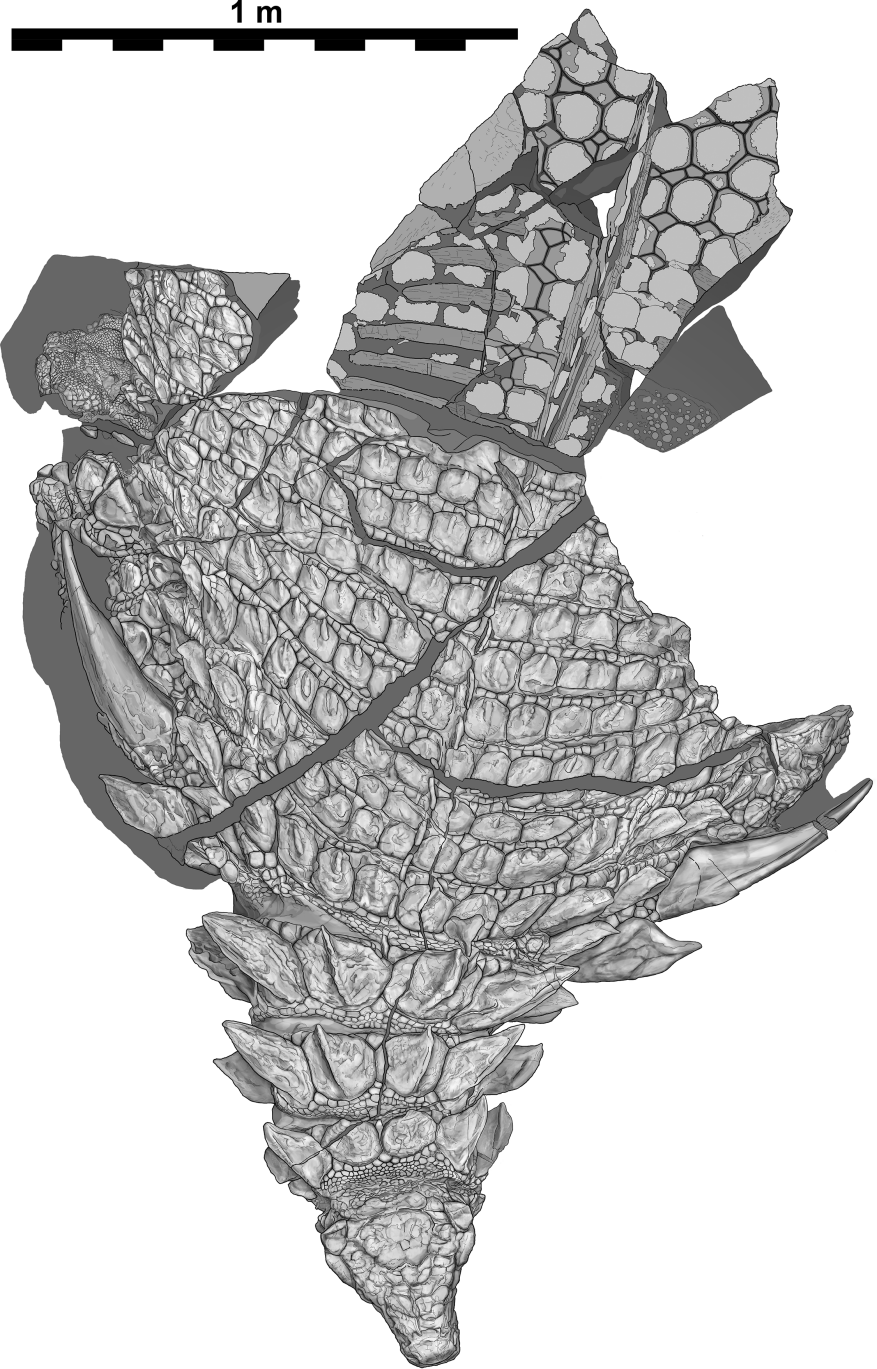
Interpretive scientific illustration of TMP 2011.033.0001 in dorsal view. Sacral region represents original part—reflected counterpart shown in Fig. 4. Scale equals 1 m.

**Figure 4:**
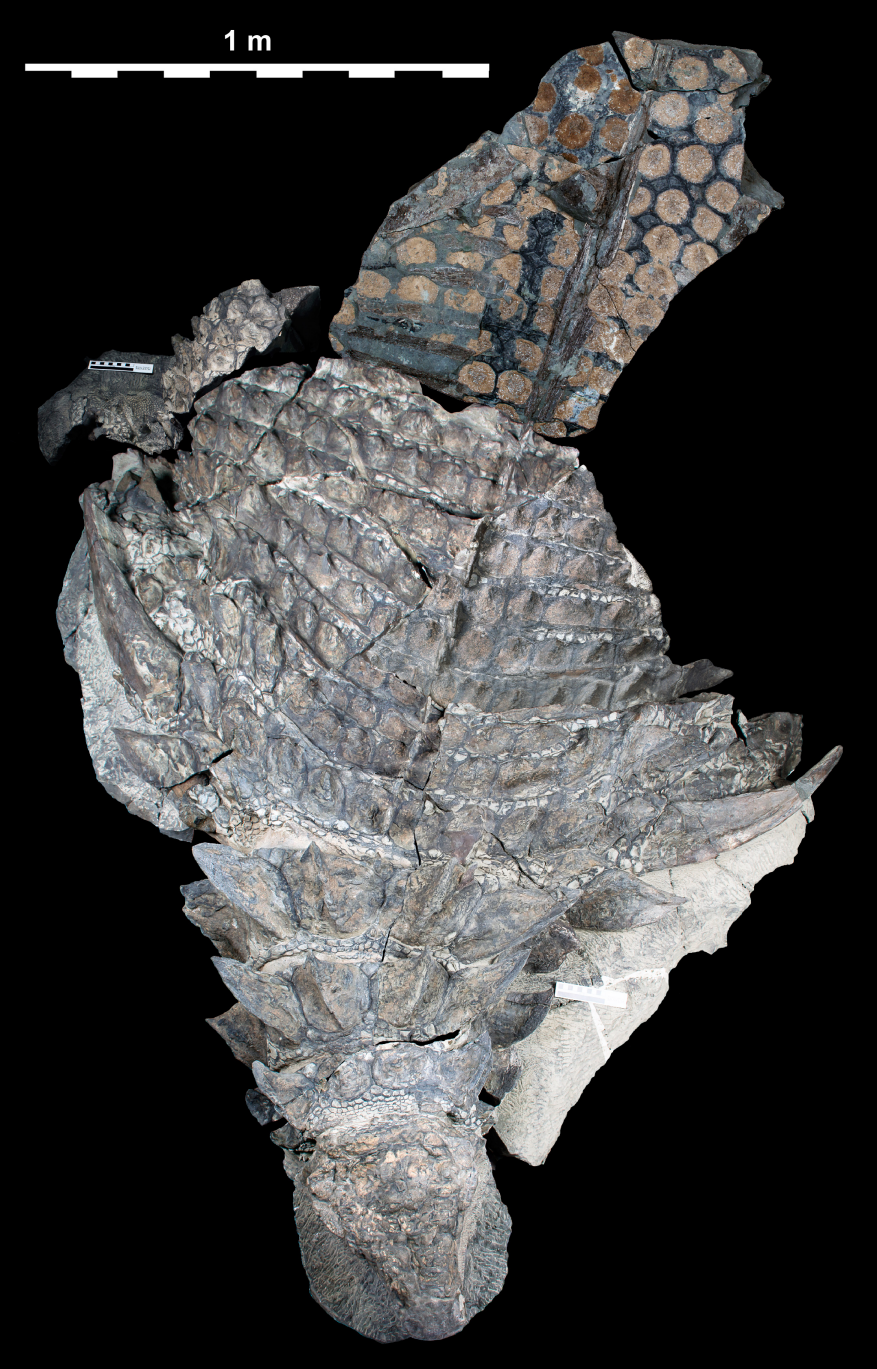
Composite dorsal view of TMP 2011.033.0001. Photocomposite created using separate images of blocks A–C, D, E, F–I, and J (see Fig. 1) and combined digitally to both reduce parallax and remove gaps. Blocks F, G, H and I represent reflected counterpart of sacral part in Fig. 2. Photographs of individual blocks were digitally modified (brightness, contrast, etc.) to removed different lighting conditions, and to illustrate an average composite of the entire specimen. Scale equals 1 m.

The specimen under examination is TMP 2011.033.0001, the holotype of *Borealopelta markmitchelli*, an exceptionally preserved nodosaurid from the Wabiskaw Member of the Clearwater Formation (Albian aged) from northern Alberta (Brown et al., 2017). The specimen preserves nearly complete and uninterrupted dermal and epidermal coverings across the anterior, dorsal and lateral surfaces of the skull; dorsal and lateral surfaces of the neck, and thoracic regions, dorsal aspect of the sacrum, lateral and posterior surfaces of the left and right forelimb, palmar surface of the right forefoot, and plantar surface of a hind foot (Figs. 1–4). These dermal and epidermal components are preserved in the form of bone for the osteoderms, and dark organic residues for the epiosteodermal and basement scales. Importantly, and distinct from the general situation in which organic epidermal residues are preserved, the specimen maintains its original three-dimensional shape, with minimal compression (Brown et al., 2017).

**Figure 5:**
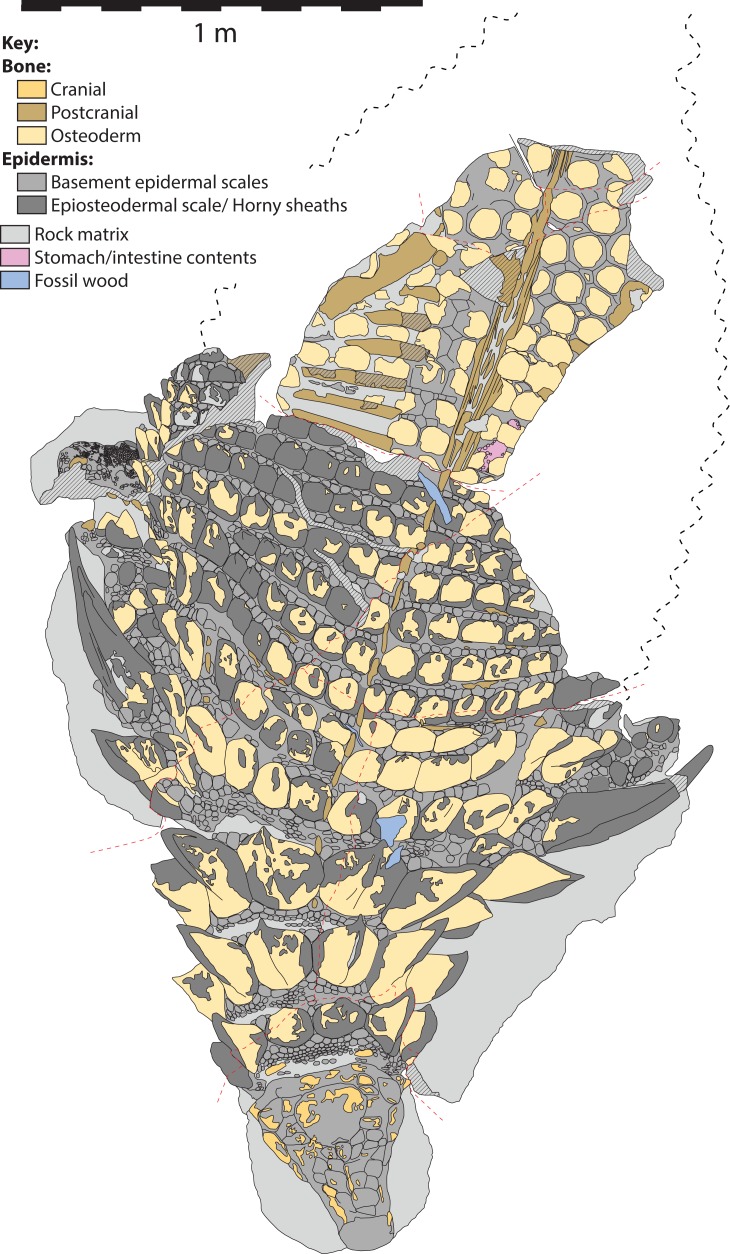
Schematic line drawing of TMP 2011.033.0001 in dorsal view, with color coding illustrating different rock and tissue types. Proportions based on Fig. 4. Scale equals 1 m.

**Figure 6:**
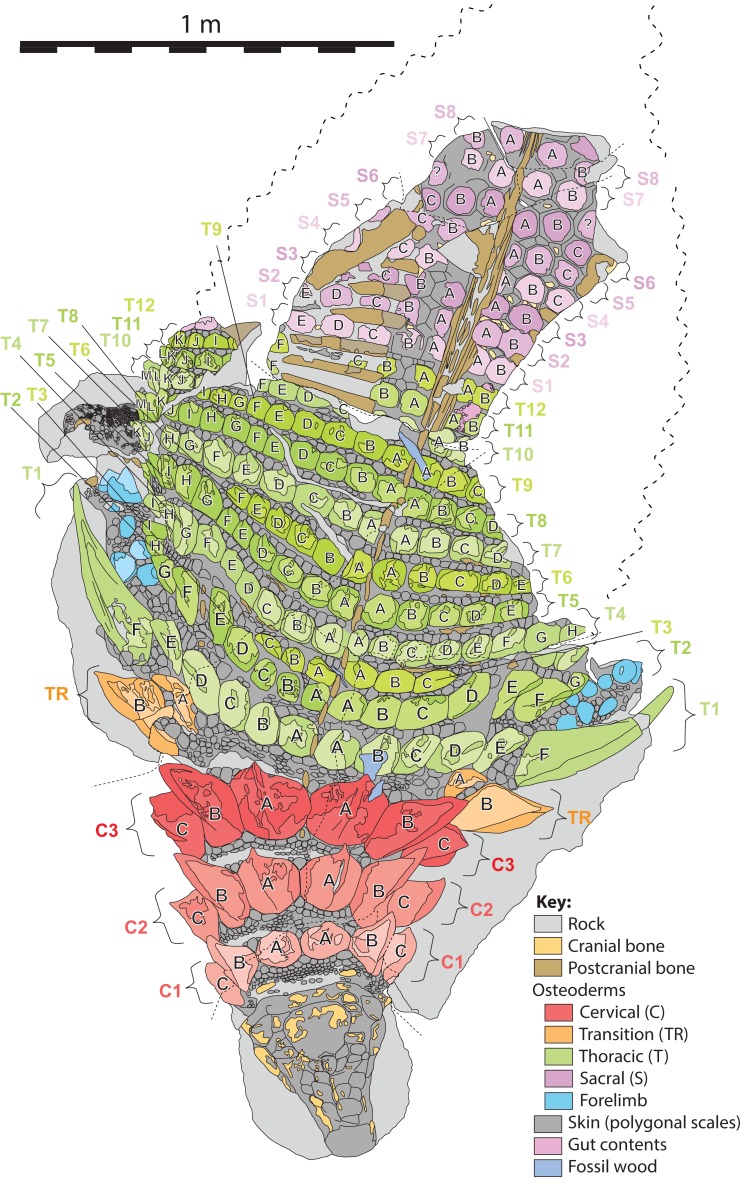
Schematic line drawing of TMP 2011.033.0001 in dorsal view illustrating osteoderm nomenclature scheme. Colors illustrate osteoderm regions (cervical, transitional, thoracic, sacral, forelimb), and transverse rows (1, 2, 3…), with letter labeling (A, B, C…) indicating position from dorsal midline. Proportions based on Fig. 4. Scale equals 1 m.

The *in situ* (i.e., in anatomic positon) axial osteoderms form distinct transverse bands that occur along the entire precaudal length of the body (Figs. 2–5). Following Burns & Currie (2014) these transverse osteoderm bands can be divided into three distinct regions: cervical: three complete bands; thoracic: 12 bands (two of which are expressed medially but pinch out laterally); and sacral: at least eight bands (Fig. 6). The boundary between the thoracic and sacral bands is estimated from the position of sacral ribs, and further preparation/scanning my change this interpretation. An additional band (expressed laterally, but pinching out medially) is located between the cervical and thoracic regions, and is termed the transitional band. No distinct caudal osteoderms are preserved, but it is possible that some of the posteriormost sacrals may turn out to belong to the base of the tail. Although osteoderm counts within these transverse bands vary greatly between these broad regions, there are areas in which these counts are consistent across several subsequent bands. As a result, consistent longitudinal lines of osteoderms are also discernable within some regions, in addition to the more obvious transverse lines.

In order to document the measurements of individual osteoderms, a nomenclatural scheme (or grid), which identifies elements by a combination of their longitudinal and transverse position, is required. Previous schemes for documenting osteoderm position have been proposed (Blows, 2001; Burns & Currie, 2014; Ford, 2000), but these systems were devised based on smaller areas of preservation, and do not scale easily when the number of osteoderm bands and/or osteoderms increases, due to preservation or taxonomy, and therefore require further elaboration.

Herein, the position of individual axial osteoderms is referred to by firstly their region (cervical, transitional, thoracic, sacral), followed by transverse band (or “row”), followed by position from the dorsal midline (reflecting rough anterior–posterior lines—hereafter termed “columns”), and finally the side of the body (Fig. 6). For convenience, the transverse bands are ordered numerically from anterior to posterior (1, 2, 3, …) (but see Burns & Currie, 2014; Ross & Mayer, 1983) within each region, and the distance from the dorsal midline (reflecting longitudinal columns) is ordered alphabetically (A, B, C,…) from medial to lateral. Under this scheme, the medialmost osteoderm (A) in the second (2) cervical (C) band on the right (R) side is designated C2AR. See Fig. 6 for a schematic illustrating the numbering scheme across the preserved specimen. This numbering scheme is used internally in an attempt to precisely designate individual osteoderms for both morphometric and descriptive purposes.

This numbering scheme is designed to be general enough to be applicable to other ankylosaurian taxa for which suites of osteoderms are preserved in the original anatomical position (e.g., *Edmontonia*—AMNH 5665, *Sauropelta*—AMNH 3036, *Scolosaurus*—NMHUK R5161, and *Kunbarrasaurus*—QM F18101). It is intended primarily for clear identification/designation of individual osteoderms, and is not intended to necessarily imply homology between taxa if applied in a broader taxonomic context, nor serial homology between osteoderm regions of a single specimen. However, it is likely that within specific regions, osteoderms within particular longitudinal columns, across transverse bands, represent serial homologues (e.g., cervical half-rings). It should be noted that both the alphanumeric designation, and directionality of the sequences are opposite the uses of Blows (2001) for the sacral shield of *Polacanthus*.

### Morphometrics and allometry

#### Osteoderms

**Figure 7:**
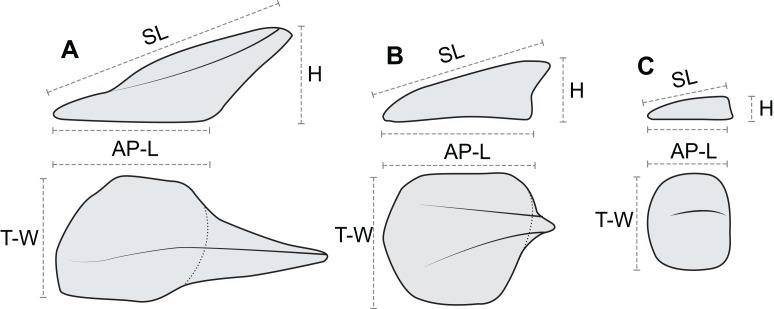
Linear osteoderm measurements used in allometric and morphometric analyses. Schematic drawing illustrating the four linear measurements obtained for representative osteoderms with (A) well-developed spines, (B) moderately developed spine, and (C) medial ridge. AP-L, anteroposterior length; T-W, transverse width; H, height; SL, spine length. AP-L, T-W, and H are mutually orthogonal and rectilinear.

In order to document and analyze variation in osteoderm shape and size, each osteoderm (where possible) was measured for four linear morphometric variables (Fig. 7). The maximum length of the base (footprint) in the anteroposterior (parasagittal) plane—“anteroposterior length (AP-L).” The maximum width of the base (footprint) in the transverse plane—“transverse width (T-W).” The maximum height of the osteoderm from the base to the apex (perpendicular to the plane of the base)—“height (H).” The maximum length of the osteoderm, from the anterior margin of the osteoderm to the apex of the spine—“spine length (SL).” Measurements for AP-L, T-W, and height, are all mutually orthogonal, whereas spine length generally represents the longest measurement in any axis. For osteoderms without a prominent keel or spine, the AP-L and SL will be the same or similar. All measurements were taken to the nearest millimeter, using a digital caliper for measurement less than 150 mm, a dial caliper for measurements between 150 and 300 mm, and a flexible fiberglass tape for measurement greater than 300 mm. Due to the inconsistency in the preservation of the keratinous coverings of the osteoderms, and occasionally indistinct boundaries between tissue types, the measurements incorporate a mixture of measure of the bony core in some instances, and the keratinous coverings in others. This will undoubtedly add higher variation to the dataset overall, as well as additional sources of error. Future internal scanning of the specimen may allow for reducing this source of error, but until this is possible the uncertainty is inherent in the dataset. A total of 172 *in situ* (in anatomical position) axial osteoderms were measured for 605 measurements in this manner (Table S1). Although appendicular osteoderms are preserved across the forelimbs, these were not included due to small sample size and/or indistinct three-dimensional nature. Very slight dorsoventral compaction of the specimen has likely occurred, and this may affect some measurements, especially across different tissue types. Despite this, the measurements analyzed here have not be adjusted to compensate for any overall, or differential, compaction, which may represent an addition source of error.

#### Epiosteodermal scales/horny sheaths

Although the preservation of most osteoderms allows for only documenting the dimensions of either the bony core, or horny sheath, but not both, a small number of osteoderms are preserved in such a way that allows for these measures to be determined independently. In total, 24 osteoderms for which both lengths of the bony core, and length of the horny keratinous sheath could be obtained, were documented (Table S2).

## Analyses

Values were log transformed (base 10) in order to reduce the effect of extremes, and to bring the distribution closer to that of a normal distribution. Bilateral data (those derived from both left and right sides) were treated in three different ways. Firstly, the data for left and right pairs were kept separate and analyzed independently. Secondly, the data for the left and right sides were averaged between paired elements. Thirdly, the data for right and left sides were pooled and treated is though derived from independent sources.

Data management and analyses were performed in Microsoft Excel (V12.3.6) and the R statistical language (V3.1.2) (R Development Core Team, 2009). Variation was quantified using standard deviations, allowing for comparisons between transverse rows and regions. Regressions analysis, both for ordinary least squares (OLS) and standardized major axis (SMA), was performed using the package “*smatr*” (Warton et al., 2011, 2012). Normality tests were conducted utilizing the Shapiro–Wilk normality test in the “*stats*” package.

In order to visualize the multivariate shape differences within and between osteoderms, transverse bands, and regions, the multivariate data were subjected to principal component analysis (PCA), allowing for the multivariate date to be represent on a fewer number of axes. The vast majority of the preserved pre-sacral osteoderms could be measured for all four linear metrics, however complete measures for several osteoderms were not possible due to incompleteness or obscuring matrix/soft tissue. To accommodate the missing values, the 172 osteoderm dataset was culled to 142—largely removing the sacral osteoderms preserved as part and counterpart. Two width occurrences were still missing in the dataset and these were estimated via multiple imputation using the “*mice*” function in the package “*mice*” (Buuren & Groothuis-Oudshoorn, 2011), see Brown, Arbour & Jackson (2012). PCA was conducted using the “*princomp*” function. Linear discriminate analysis, using the function “*lda*” in the package “*MASS*” (Ripley et al., 2015) was used to test the ability to categorized individual osteoderms to groups (complete specimens only), based on the centroids of their respective groups (Hammer & Harper, 2006; Venables & Ripley, 2002).

## Results

### Size and variation

**Figure 8:**
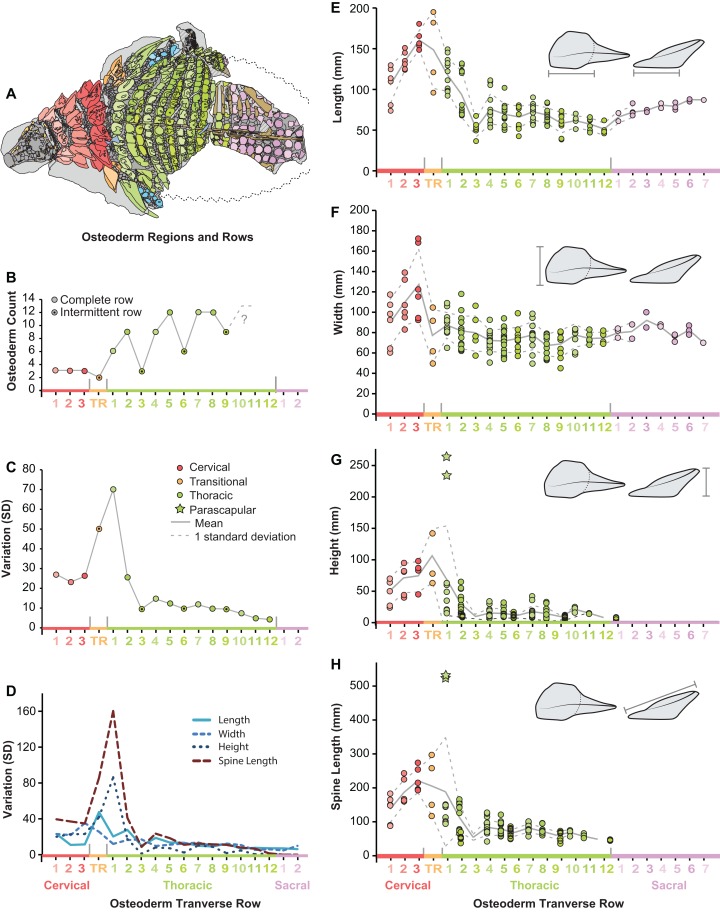
Plots illustrating osteoderm count, variation, and linear measurements across the transverse osteoderm rows. (A) Line drawing illustrating color coding of osteoderm regions and transverse rows in TMP 2011.033.0001 (see Fig. 6). (B) Osteoderm count for each transverse row. (C) Total variation within each osteoderm row. (D) Variation of each linear metric within each osteoderm row. Raw measures of osteoderm anteroposterior length (AP-L) (E), transverse width (T-W) (F), osteoderm height (H) (G), and total spine length (SL) (H). Stars in (G) and (H) indicate outlying parascapular spine morphs.

Within the entire preserved osteoderms series, distinct patterns of osteoderm size and shape are seen both longitudinally (anteroposteriorly) and transversely (mediolaterally). Within the cervical region, all variables increase in size steadily from the anteriormost to posteriormost band (Figs. 8E–8H). Basal length and width rapidly decrease across the transitional band and into the thoracic regions, where the values remain relatively constant throughout the series (T3–T12) (Figs. 8E and 8F). Although not well preserved, osteoderm basal length and width appear to increase again within the sacral series. As with measures of the osteoderm basal footprint, osteoderm height and spine length also decrease into the thoracic region, however the first several bands (TR and T1) include individuals (indicated by stars) that eclipse all anterior values in magnitude (Figs. 8G and 8H). Unlike basal length and width, values for height and spine height were unable to be scored for the sacrals due to preservation.

Across the pre-sacral osteoderm series, the highest amount of morphological variation (size and shape) is seen in the cervical region, transitional band, and anteriormost thoracic band, with low variation seen in most posterior thoracic bands T3–T12 (Fig. 8C). This overall pattern of variation is not uniform between the different linear metrics, rather variation within these measures show slight differences within the series (Fig. 8D). Variation in the basal footprint of the osteoderms is high in the cervical bands, but low for the thoracic bands (Fig. 8D). Contrasting this, the high overall variation in the anteriormost thoracic band (T1) is due, almost exclusively, to osteoderm height and spine length, and mainly driven by the large and elongate parascapular spine (Fig. 8D).

### Osteoderm regression and allometry

Analyses of correlation and regression of bivariate pairs indicates that the different treatments of left/right pairs (independent, averaged, or pooled) has only a marginal effect on the results (Table 1). Although absolute correlation and regression parameters are affected by the differing treatments of pairs, in no cases was the significance of the correlation, nor the scaling trend (allometry vs. isometry) affected. For convenience, the values reported below are derived from the averaged analyses. All four variables are significantly correlated, but the strength of the relationship varies greatly. Osteoderm basal length and osteoderm spine length have the strongest correlation (*R*^2^ = 0.85), followed by osteoderm height and spine length (*R*^2^ = 0.82) (Table 1). The weakest correlation is seen between osteoderm width and osteoderm height (*R*^2^ = 0.12).

**Table 1:** Regression analysis of *in situ* osteoderms.

			Correlation		Regression (OLS)				Regression (RMA)			
*X*	*Y*	*N*	Sig	*R*^2^	Slope	LCI	UCI	Allo	Slope	LCI	UCI	Allo
**Left/right averaged**												
AP-L	TW	97	2.58E–07	0.23	0.29	0.19	0.39	Neg	0.59	0.49	0.70	Neg
AP-L	H	93	2.20E–16	0.67	1.82	1.56	2.08	Pos	2.22	1.97	2.50	Pos
AP-L	SL	93	2.20E–16	0.85	1.21	1.11	1.31	Pos	1.31	1.21	1.42	Pos
TW	H	94	2.81E–04	0.12	1.35	0.64	2.06	Iso	3.71	3.06	4.49	Pos
TW	SL	93	1.14E–06	0.22	1.06	0.66	1.46	Iso	2.22	1.85	2.66	Pos
H	SL	92	2.20E–16	0.80	0.53	0.47	0.58	Neg	0.59	0.54	0.65	Neg
**Left/right pooled**												
AP-L	T-W	158	2.10E–10	0.22	0.30	0.21	0.38	Neg	0.62	0.54	0.72	Neg
AP-L	H	141	2.10E–10	0.69	1.89	1.68	2.10	Pos	2.28	2.08	2.50	Pos
AP-L	SL	141	2.20E–16	0.84	1.23	1.14	1.32	Pos	1.34	1.25	1.43	Pos
T-W	H	140	1.63E–06	0.15	1.43	0.87	2.00	Iso	3.66	3.14	4.27	Pos
T-W	SL	139	7.34E–09	0.21	1.01	0.68	1.33	Iso	2.16	1.87	2.51	Pos
H	SL	141	2.20E–16	0.82	0.53	0.49	0.57	Neg	0.59	0.55	0.63	Neg
**Left/right independent**												
**Left**												
AP-L	T-W	57	3.50E–03	0.13	0.24	0.08	0.39	Neg	0.63	0.49	0.80	Neg
AP-L	H	48	7.47E–16	0.75	2.27	1.89	2.66	Pos	2.62	2.27	3.03	Pos
AP-L	SL	48	2.20E–16	0.86	1.34	1.18	1.50	Pos	1.45	1.30	1.62	Pos
T-W	H	48	5.66E–03	0.13	1.67	0.51	2.82	Iso	4.28	3.28	5.59	Pos
T-W	SL	48	2.91E–03	0.16	0.98	0.35	1.62	Iso	2.36	1.82	0.08	Pos
H	SL	48	2.20E–16	0.87	0.52	0.46	0.57	Neg	0.55	0.50	0.61	Neg
**Right**												
AP-L	T-W	87	1.99E–07	0.26	0.32	0.21	0.44	Neg	0.62	0.52	0.75	Neg
AP-L	H	86	2.20E–16	0.65	1.74	1.47	2.01	Pos	2.16	1.90	2.45	Pos
AP-L	SL	86	2.20E–16	0.81	1.17	1.05	1.28	Pos	1.29	1.18	1.41	Pos
T-W	H	85	3.25E–04	0.13	1.29	0.61	1.98	Iso	3.41	2.80	4.17	Iso
T-W	SL	84	4.79E–06	0.21	0.98	0.58	1.37	Iso	2.36	1.82	3.08	Iso
H	SL	86	2.20E–16	0.79	0.53	0.47	0.59	Neg	0.60	0.54	0.66	Neg

**Note:**

Regression, both ordinary least squares and reduced major axis regression) based on four linear metrics, with three different treatments of bilateral data (averaged, pooled, and independent). For data see Table S1.

Irrespective of the regression model, osteoderm width is negatively allometric, while height and spine length are positively allometric, with respect to the osteoderm parasagittal length (Table 1). Osteoderm height and spine length are both positively allometric with respect to width under SMA, but isometric under OLS. Osteoderm height is also negatively allometric with respect to spine length across both regression models. Relative to the osteoderm width, the allometric coefficients of length, spine length, and height are 1.60, 2.16, and 3.66, respectively (Table 1). Taken together these results illustrate that as the osteoderm bases increase in size (regionally) aspects of osteoderm height and spine length increase at a much higher rate.

**Figure 9:**
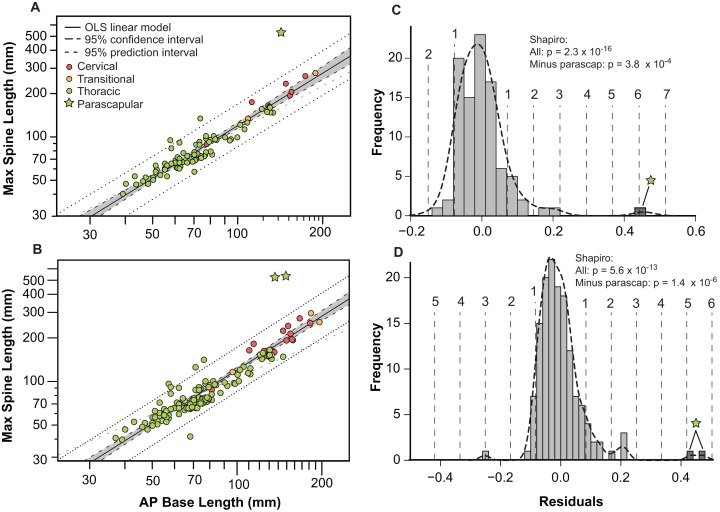
Allometry of osteoderms. Spine length (SL) regressed as a function of anteroposterior basal length (AP-L) for bilaterally averaged (A) and bilaterally independent (B) osteoderms. Colors pertain to osteoderm region (see Fig. 6). Ordinary least squares regression indicated by solid line (Table 1), shaded area indicates 95% CI of the relationship, and dotted lines indicate 95% predication interval of points. Plots (C) and (D) illustrate histograms of the residuals of (A) and (B) (respectively) with vertical dotted lines indicating standard deviations. Stars indicate outlying parascapular spines.

Bivariate plots indicate that the different osteoderm metrics co-vary in consistent ways, regardless of the different body regions, and positions (Fig. 9). However, the exception to this pattern are the large, parascapular spines. When the length of the osteoderm spine is regressed against the parasagittal length of the osteoderm base, the resulting regression is tight and positively allometric, but with the parascapular spines consistently falling outside (well above) the 95% prediction intervals (Figs. 9A and 9B). This indicates that the parascapular spines show a distinctly stronger pattern of allometric scaling in spine length than all the other osteoderms. This is also clearly illustrated when a histogram of the regression residuals is plotted, both for averaged (Figs. 9A and 9C) and pooled (Figs. 9B and 9D) sides. In both cases, the parascapular spines are the strongest outliers, double those of any other point, and located at least five standard deviations from the best fit line.

### Epiosteodermal scale/sheath contribution and allometry

Across the entire pre-caudal series, the basal footprint of the epiosteodermal scale (sensu Arbour et al., 2014) closely matches the basal footprint of the underlying osteoderm, both in terms of size and shape. Contrasting this, the contribution of the epiosteodermal scale to topography of the osteoderm, specifically the height and overall spine length, is highly variable between the different regions. For the majority of the thoracic osteoderms (rows T3–T12), the epiosteodermal scale is a relatively uniformly thick layer capping the entire apical surface of the osteoderm. This layer is generally between 3 and 5 mm thick and makes up between 2% and 6% of the overall (i.e., combined osteoderm and epidermal cover) height. In the anteriormost thoracic osteoderms (T1–T2), but excluding the parascapular spine, the epiosteodermal scale is of similar thickness across the flattened portion of the scale, but increases in thickness toward the keel, with the thickened portion running along the apex of the keel. This thickened epiosteodermal keel ranges in thickness from 9 to 17 mm, making up 6–13% of the overall height.

The expression of the epiosteodermal scale on the transitional and cervical osteoderms is similar to, but more exaggerated than, that of the anterior thoracics. Here the thickness of the epiosteodermal scale on the flattened (basal) portions of the osteoderm, and the gently sloping flanks, is relatively moderate (2–6 mm), but scale thickness increases dramatically in association with the underlying bony keel, forming a horny keel ranging from 10 to 48 mm in thickness. These horny ridges form along both the entire anterior (leading) and posterior (trailing) edges of the bony keels (increasing its apparent profile), but also project posterior to the posteriormost apex of the spine—greatly increasing the apparent apical length/height of the spine, and making up 9–20% of the overall length. In these extreme cases, due to the conical shape of the bony core and the hollow cone shape of the overlaying epiosteodermal scale, the term “sheath” may better describe these epidermal structures than “scale.”

**Figure 10:**
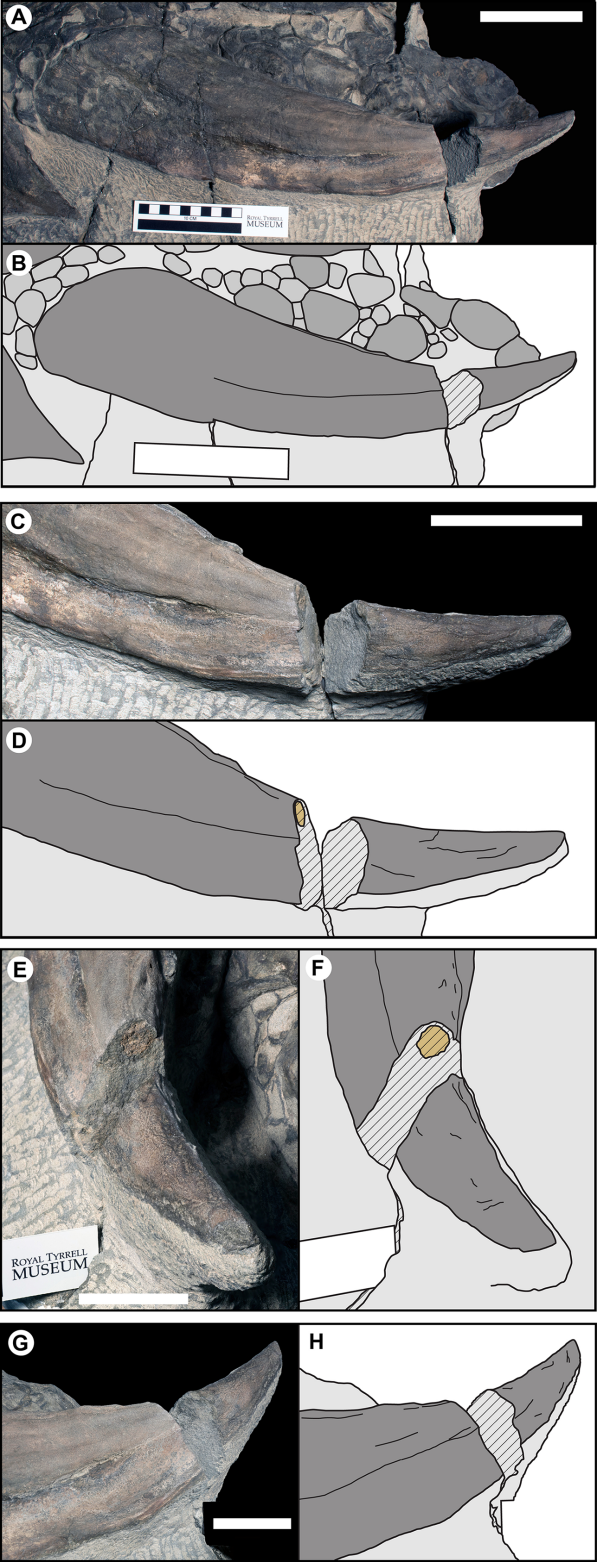
Photographs and line drawing of the left parascapular spine (T1FL) of TMP 2011.033.0001, showing the morphology of the keratinous sheath and bony core. (A and B) Dorsal view of complete left parascapular spine. (C and D) Close up (dorsal view), showing the distal break in the spine, with bone core proximal and not bone core distal. (E and F) Distal (dorsolateral) view of break, showing proximal cross-section surface with bony core. (G and H) Proximal (dorsomedial) view of break, showing distal cross-section surface without bony core. Color code same as Fig. 3. Scale bars equal 10 cm for (A–D), and 5 cm for (E–H).

The parascapular spine (T1F), bears the most extensive contribution of the epiosteodermal covering. On both the left and right sides the dorsal surface of the bony osteoderm is obscured by a nearly continuous, thick (3–10 mm), epiosteodermal covering. A fortuitous break, three-quarter of the distance to the apex, on the left parascapular spine exposes the cross-section (Fig. 10). The break results in a 37 mm gap in the spine, with the distal cross-section 117 mm from the apex and the proximal cross-section 154 mm from the apex. The proximal cross-section reveals a small circular bony core, ∼15 mm in diameter (Figs. 10C–10F). This bony core is not present on the distal cross-section (Figs. 10G and 10H), indicating it terminates between 117 and 154 mm from the apex of the sheath, with the final 117–154 mm of the spine composed of only the horny sheath (midpoint = 25% of the total length, or a 133% increase in apparent length). The cross-section of the bony core is not positioned centrally within the preserved sheath, but rather is restricted to the posterior one-quarter of the 60 mm diameter horny cross-section. The anterior two-thirds of the sheath is completely unsupported by bone, and is noticeably ventrally depressed, and bearing a more rugose texture (Fig. 10). This is consistent with a pattern seen on both sides, where the anterior margin of the sheath 27–39 mm wide is ventrally depressed and differently textured than the posterior portion (Figs. 3 and 10). This region is of relatively consistent breath across its length, but tapers slightly toward the base. Taken together the cross-section and depressed anterior margin indicate a thin, but broad (∼30–40 mm wide) flange of keratinous sheath extending along the entire length of the leading edge of the parascapular spine. Overall, the keratinous portions of the parascapular spine greatly increase the apparent size of the spine, with the anterior flange of the sheath increasing the width by a factor of 2–3×, and the keratinous tip increasing the length by a factor of ∼1.3× (Fig. 10).

**Figure 11:**
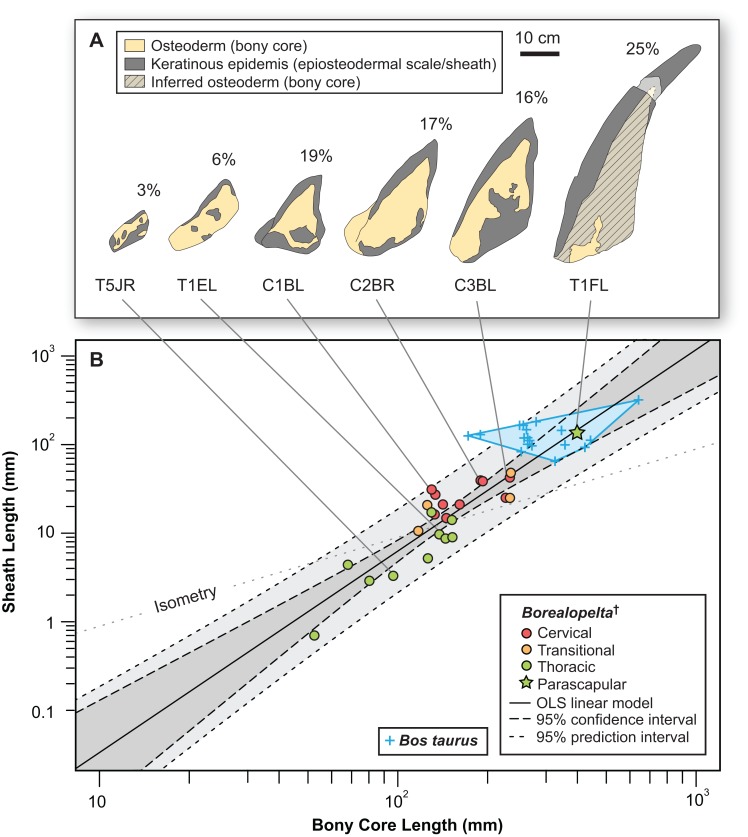
Allometry of keratinous sheath as a function of bony core length across the osteoderm series. (A) Schematic line drawing of representative osteoderms in (B), showing the relative contribution of the keratinous sheath. Numbers above each osteoderm indicate the percentage of the total length formed by keratin only. Spines T1EL, C1BL, C3BL and T1FL reflected. (B) Keratinous-only sheath length (i.e., the portion projecting beyond bony core) regressed as a function of osteoderm bony core length. Ordinary least squares regression indicated by solid line (Table 2), shaded area indicates 95% CI of the relationship, and dotted lines indicate 95% predication interval of points. Colors pertain to osteoderm region (see Fig. 6), and stars indicates parascapular spine. Blue “+” and corresponding convex hull, indicated range of relationship between bony core length and keratinous sheath length in a sample of domestic cattle horns.

Overall, when the osteoderms bear distinctive keels, the thickness of the epiosteodermal scale increases at the keel, further exaggerating the apex of the ridge (Fig. 11A). The greater the height of the bony keel, the greater the thickness of the epiosteodermal scale along that keel. In order to quantify this pattern of relative scaling of the horny coverings, the contribution of the total spine length from the epiosteodermal scale is regressed against that of the bony core. The resulting regression is very strongly positively allometric (OLS slope = 2.28, 95% CI = 1.77–2.77; RMA slope = 2.56, 95% CI = 2.10–3.11) (Fig. 11B; Table 2). This indicates that the keratinous covering increases at greater than twice the rate of the underlying bony core.

**Table 2:** Regression analysis of keratinous sheath of osteoderms and horns as a function of the bony core.

Keratinous sheath regression	Correlation			Regression (OLS)				Regression (RMA)			
	*N*	Sig	*R*^2^	Slope	LCI	UCI	Allo	Slope	LCI	UCI	Allo
***Borealopelta***	24	2.46E–09	0.79	2.28	1.77	2.78	Pos	2.56	2.10	3.11	Pos
**Extant (individual)**	106	2.20E–09	0.93	1.42	1.35	1.50	Pos	1.48	1.40	1.55	Pos
**Extant (genus means)**	8	9.62E–05	0.92	1.39	1.02	1.77	Pos	1.44	1.12	1.86	Pos

**Note:**

Regression under both ordinary least squares (OLS) and reduced major axis (RMA). For data see Tables S2 and S3.

Although not quantitatively analyzed here, some of the preserved appendicular osteoderms also bear thickened epiosteodermal scales. In particular, a serial pair of antebrachial osteoderms are capped by a uniformly thick (4–9 mm), smooth epiosteodermal scale proximally. Both osteoderms express a distinct medial keel that projects distally, and although the epidermal scale is not preserved distally, given the preserved thickness at the base, these may also have expressed significantly thicker horny coverings distally.

### Morphospace

**Figure 12:**
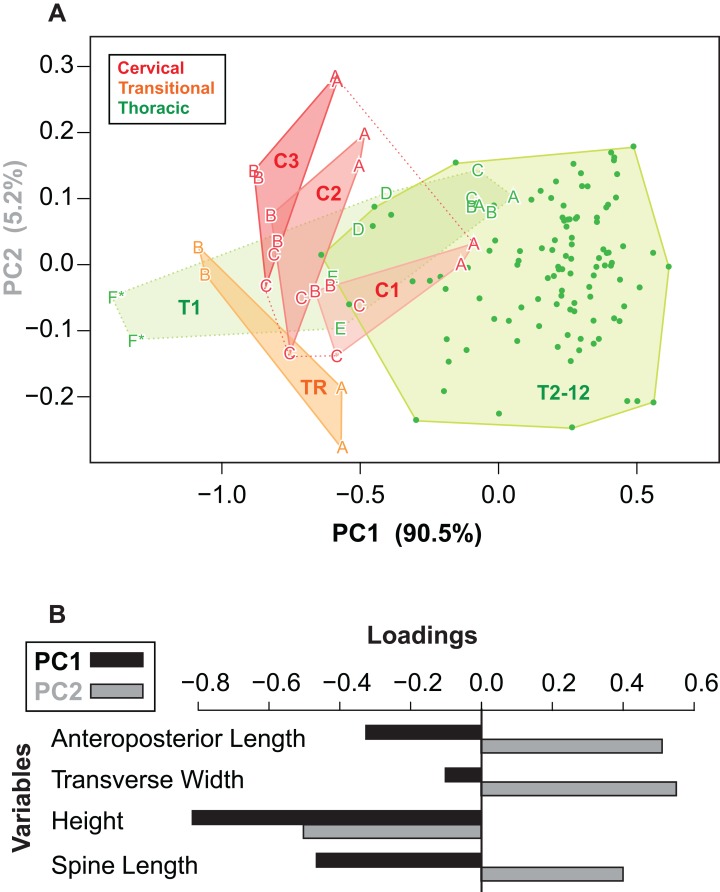
Osteoderm morphometrics. (A) Plot illustrating PC scores for PC1 (90.5% of variation) and PC2 (5.2% of variation) resulting from a principle component analysis of 142 osteoderms (Table 3). Colors reflect osteoderm regions: cervical (red), transitional (orange), thoracic (green). Points for rows C1–T1 are illustrated by letters (see Fig. 4), while T2–T12 are illustrated by dots. Colored areas represent minimum convex hulls for each row/region (T1 separate from T2–T12). “*” Denotes parascapular spines. (B) Loading of variables for principal component analysis in (A).

Given the relatively consistent pattern of size and shape variation amongst the osteoderms from different regions and positions, it may be possible to visualize these in multivariate morphospace. Following a PCA, a plot of the principal component scores for axis 1 and 2 (90.5% and 5.2% of the variance, respectively) illustrates the vast majority of the morphological variation in this sample (Fig. 12A). Component one (PC1) largely describes absolute size of the osteoderms across all variables—negatively loaded (though unequal), whereas component two (PC2) largely reflects osteoderm height, negatively loaded, relative to the remaining variables, positively loaded (Fig. 12B; Table 3).

**Table 3:** Loadings for principle component analysis of osteoderms.

	PC1	PC2	PC3	PC4
**AP-L**	−0.326	0.515	0.371	0.7
**T-W**	−0.103	0.56	−0.822	NA
**H**	−0.817	−0.507	−0.247	0.124
**SL**	−0.465	0.405	0.355	−0.703
**Prop. var.**	0.91	0.05	0.03	0.01
**Cum. var.**	0.91	0.96	0.99	1.00

**Note:**

Variable loadings and proportion of variance explained by the four different principle components.

Reasonably distinct morphospace occupation, and segregation, of osteoderms from the different regions of the body is shown (Fig. 12A). With the exception of the medial most osteoderm in the anterior cervical band (C1A), there is minimal overlap between cervical osteoderm morphospace, and that of either the transitional and thoracic regions. The relative position of the osteoderms of cervical bands 1, 2, and 3 are positioned similarly, but largely separated out on PC1. This reflects the similarity in the pattern of osteoderms across these three bands, with the major difference being the increase in size posteriorly. Osteoderms of the transitional band (TR) do not overlap with those of either the cervicals or the thoracics. The parascapular spine (T1F) occupies a distinct area of morphospace from all other osteoderms, and is separated from the remaining thoracic osteoderms by the cervical and transitional osteoderms. Aside from the parascapular spine (T1F), and to a lesser extent rest of the anteriormost band (T1), the thoracic osteoderms (T2–T12) are relatively closely spaced—suggesting low morphological disparity within these elements.

**Figure 13:**
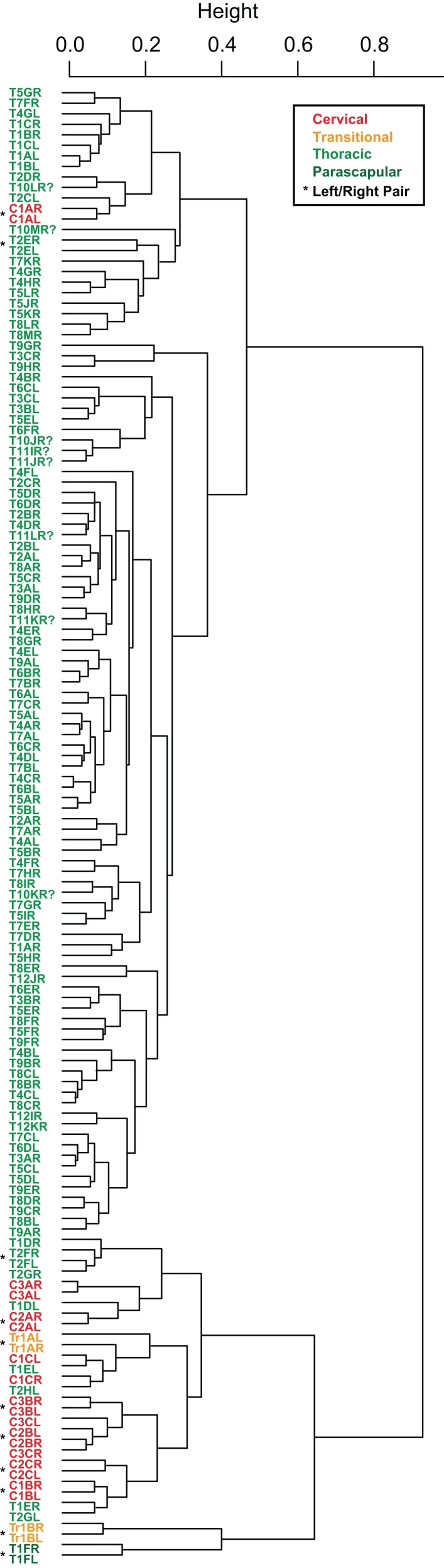
Cluster analysis of osteoderms. Cluster dendrogram of nodosaur osteoderms based on PC scores, under “average” (UPGMA) agglomeration method. Colors denote regions while “*” denotes left/right pairs.

Cluster analyses of the PC scores indicates variable results, dependent on body region. The agglomeration algorithm resulting in a dendrogram with the most anatomically consistent pattern (*a posteriori*) was average linkage (UPGMA) (Fig. 13), with other methods performing variably poorer. The results suggest successful clustering only at the coarsest (regional), and occasionally, finest (left/right pair) scales, with poor performance on the scale of row and column. The most inclusive dichotomy divides a group consisting of most cervical, all transitional, the parascapular spines, and several thoracic from the remaining thoracic osteoderms. Despite this course regional clustering, obvious clusters of the same row or column are absent. At the finest scale, however, left/right pairs for all transitional, the parascapular spines, and 78% of cervical osteoderms were recovered as either exclusive groups or in a sister positions to a more exclusive group. Contrasting this, only 5% for thoracic osteoderms (excluding parascapular spines) were correctly clustered into left/right pairs.

Despite the relatively clear separation of osteoderms by region in morphospace and cluster analysis, linear discriminate analysis was only able to correctly categorize 89% of the osteoderms into their correct anatomical region—three unequal categories: 50% for cervicals, 75% for transitionals, and 96% for thoracics (Table 4). All of the cervicals from the anteriormost band (C1) were assigned as thoracics—likely due to small size, while the lateralmost osteoderm in the second row (C2C), and one from the third row (C3C), were regarded as transitional—likely due to elongate spines. Only one transitional osteoderm was incorrectly categorized (TR1AR), which was designated a thoracic. Three anterior thoracic osteroderms (T1DR, T1DL, and T1ER) are categorized as cervical, while the left parascapular spine (T1FL) was categorized as a transitional. Linear discriminate analysis based on transverse rows (16 categories) and longitudinal columns (13 categories) indicates the ability to correctly classify osteoderms into subgroups does not persist at scales smaller than the three broad regions, with only 13% and 22%, of osteoderms categorized correctly by row or column, respectively.

**Table 4:** Results of linear discriminate function analysis.

Region	Assigned			
	Cervical	Transitional	Thoracic	Proportion correct
**Cervical**	***9***	3	6	0.5
**Transitional**	0	***3***	1	0.75
**Thoracic**	4	1	***113***	0.96

**Note:**

Rows indicate correct placement of osteoderms into regions and column indicate assignment via linear discriminate function analysis. Values in the diagonal, bold and italic, represent correct assignment.

## Discussion

### Osteoderm variation and allometry

The morphometric analysis of a nearly complete pre-sacral osteoderm series from an exceptionally preserved nodosaurid has produced quantitative results both supporting previous qualitative suggestions, and novel inferences regarding the scaling and variation of these structures. Across the preserved specimen, the osteoderms with the highest level of morphological variation or disparity are those positioned from the cervical to the anterior thoracic regions. This is evidenced by three distinct metrics; (1) higher levels of within-band variation (Figs. 8C and 8D), (2) more dispersed morphospace occupation (PC scores) within regions (and between elements) (Fig. 12A), and (3) and better clustering and much higher frequency of correct clustering of left/right pairs (Fig. 13). This variation is particularly high across the cervical to thoracic transition (C3, TR, and T1). In contrast to the anterior portion of the animal, the posterior thoracic bands (T2–T12) show remarkably little variation, both within and between transverse bands (Figs. 8C and 8D, 12A). Taken together, these results suggest a cephalization in the morphological disparity of the osteoderms, with posterior thoracis showing very low variability. This effectively quantifies the general qualitative pattern seen in many nodosaurids, with high size/shape variation of cervical and pectoral osteoderms, but a more consistent pattern in their posterior counterparts, e.g., *Sauropelta* (Carpenter, 1984).

The high within specimen disparity within the cervical and pectoral osteoderms is intriguing as these regions are also where the highest intraspecific variation and the most taxonomically useful species-specific armor morphology occurs in both Nodosauridae and, potentially, Ankylosauridae (Blows, 2014; Carpenter, 1990; Penkalski, 2001), though see taxonomic utility of ankylosaurid caudal and pelvic armor (Arbour & Currie, 2013a, 2013b; Arbour & Evans, 2017). This is similar to the situation in Hadrosauridae and Ceratopsidae, where the crests, horns and frills, are the most variable regions, both within individual and between specimens, and also the regions that are the most useful for species-level taxonomy (Currie, Langston & Tanke, 2008; Dodson, 1975, 1976; Evans, 2010). It is also of note that this highly variable and species-specific region is somewhat cephalized toward the anterior extreme of the animal. Although not located on the skull as in Hadrosauridae, Ceratopsia, and Pachycephalosauria, these structures would still have been prominent in a frontal view of the animal (Carpenter, 1982, fig. 3), representing a potential front-on visual display to inter- and/or intraspecifics. It should be noted that completeness/sampling biases may also be partly driving this pattern, as the cervical osteoderms in Ankylosauridae are fused, and more likely to be preserved in position, and therefore useful for taxonomic diagnoses.

Segregation of osteoderms from different regions in both the ordination, and the cluster analysis, but lack of segregation at the scale of transverse rows or longitudinal columns, suggests that isolated osteoderms may be confidently assigned to broad anatomical regions, but likely not beyond this level (with isolated exceptions). This suggests that for specimens where the osteoderms position has been lost (i.e., in disarticulated specimens) it may be difficult to objectively assign individual osteoderms to their original position beyond the coarsest anatomical regions based on broad shape/size data. Exceptions, such as the parascapular spine, or cervical bands likely exist.

The similarity of PC scores, and exclusive clustering of left/right pairs for some osteoderms, indicates that: (1) original bilateral symmetry within the osteoderm suite is retained in the fossil, (2) these longitudinal and transverse elements are often unique enough in their morphology that left/right pairs can be objectively determined, and (3) this morphology is effectively captured by the measurements.

It is entirely possible that alternate or more detailed approaches to osteoderm morphometrics may result in higher differentiation between osteoderms derived from distinct regions and rows/columns. These could include a higher number of linear measurements, or, more likely, a better assessment of shape from geometric morphometric, either two-dimensional or three-dimensional. Until this is achieved, objective methods for assignment of isolated or anatomically displaced (disassociated) osteoderms to specific localities is likely limited to broad anatomical regions. Additionally, if the osteoderm suites prove to be highly species-specific, results from this one taxon may have limited application for other taxa within Ankylosauria.

Allometric analysis shows that within the sampled osteoderms, their relative proportions do not change isometrically, but rather are strongly allometric. These data reflect regional allometric scaling within a sample of elements that are, at least at some scale, serially homologous and show that osteoderm spine height increases much faster than the basal footprint. Moreover, while the shape/size variation in osteoderms from all sampled regions can largely be explained by a single allometric equation, the parascapular spine is a strong outlier. The shape of this spine requires a scaling pattern distinct from the remaining osteoderms, and may reflect different drivers in its development and evolution. Although these scaling patterns are not directly equivalent to ontogenetic allometry—determined either from sampling a single individual through its lifespan (“longitudinal studies,” e.g., Cock, 1966; Jungers & Fleagle, 1980), or multiple individuals at various stages of ontogeny (“cross-sectional studies,” see Alberch et al., 1979 and citations therein; Gould, 1966)—they are pertinent to the patterns of relative scaling responsible for creating the varied osteoderm morphologies, regionally, within the animal.

### Keratinous sheaths

The projecting osseous structures of many dinosaur groups, including the osteoderms of thyreophorans, horns of ceratopsians, and domes of pachycephalosaurs, have prompted speculation regarding the form and contribution of their epidermal coverings. Although a keratinized epidermal covering is largely agreed upon (Dodson, 1996; Galton & Sues, 1983; Gilmore, 1914, 1920; Goodwin & Horner, 2004; Hatcher, Marsh & Lull, 1907; Hayashi et al., 2010; Hieronymus et al., 2009; Horner & Goodwin, 2008, 2009; Main et al., 2005; Marsh, 1880; Moodie, 1910; Penkalski & Blows, 2013; Saitta, 2015; Sampson, Ryan & Tanke, 1997; but see De Buffrénil, Farlow & De Ricqles, 1986), to what degree this covering exaggerates the length or profile of the bony core remains unknown. It is important to note that it is these keratinous coverings, not the bony cores, of the structures that either directly interact with the environment or other animals, or are perceived by inter- and conspecifics. Furthermore, work on extant Bovidae has illustrated that the bony horn core is relatively conservative across taxa, and it is largely the keratinous sheath that is responsible for the great diversity of horn morphology in this radiation (Geist, 1966). As such, understanding the morphology and relative proportions of the keratinous epidermis is important in understanding their function and evolution.

The exceptional preservation of epidermal structures in TMP 2011.033.0001 greatly expands our empirical dataset on the epidermis of these exaggerated structures within Thyreophora. Firstly, this specimen provides morphological evidence for the presence of thickened, keratinized coverings on nearly all axial and appendicular osteoderms (Brown et al., 2017). Of the 173 osteoderms exposed in apical view, 168 (97%) have preserved epidermal coverings, leaving no questions as to the ubiquitous nature of this tissue. When combined with other occurrences of more isolated preserved keratinized osteodermal coverings in basal Thyreophora (Martill, Batten & Loydell, 2000), Stegosauria (Christiansen & Tschopp, 2010), and Ankylosauridae (Arbour et al., 2013, 2014), this provides broad support that all thyreophoran osteoderms were neither naked, nor covered by thin tissue, but capped by a thickened keratinous layer. Importantly, and contrasting with these previous finds, this marks the first occurrence of a keratinous epidermis that does significantly alter and exaggerate the length/profile of the underlying osteoderm.

Secondly, the keratinous coverings are neither of consistent thickness (e.g., 5 mm), nor represent a constant proportion (e.g., 10%) of the size/height of the osteoderm. Rather the thickness of the keratinous covering is strongly positively allometric (slope = 2.2 or 2.5) with respect to the osteoderm (bony core) height, with the epidermal covering of the posterior thoracics being marginal, and the parascapular spine being ∼25% of the total length. How predictive the allometric relationship in TMP 2011.033.0001 is for the keratinous coverings in other ornithischian taxa is unknown, but it indicates that simple percentage increases—i.e., at least a 10% increase in the length of pachycephalosaur horns (Galton & Sues, 1983), are likely overly simplistic. If these results of allometric scaling of the sheaths are generally applicable across Thyreophora, one would predict that for low-profile osteoderms, e.g., across basal taxa such as *Scutellosaurus* and *Scelidosaurus*, or those of certain regions in Ankylosauridae and Nodosauridae, their keratinous coverings will be subtle. However, those osteoderms with large keels, or modified into large spines or plates—e.g., cervicoscapular spines of Nodosauridae, and dorsal spines and plates of Stegosauria—likely bear substantial keratinous sheaths, greatly increasing their apical length and/or profile. The first of these predictions appears to be consistent with previous reports (Arbour et al., 2013, 2014; Christiansen & Tschopp, 2010; Martill, Batten & Loydell, 2000; Penkalski & Blows, 2013), however the second remains untested until additional exceptionally preserved taxa are found.

Our understanding of the proportions of the keratinous spine, relative to the bony core, for this extinct animal, bears comparison with extant analogues. Somewhat ironically, however, although growth/scaling of horns (and horn-like structures) in mammals has been investigated heavily (Bergeron et al., 2008; Bunnell, 1978; Côté, Festa-Bianchet & Smith, 1998; Festa-Bianchet et al., 2004; Hardenberg et al., 2004; Loehr et al., 2007), few studies have quantified the pattern for relative growth/scaling of both the keratinous and bony portions of horn-like structures—whether in the context of ontogeny/phylogeny or elements that are serial homologous. Even more rarely are these patterns documented in extant squamates and/or birds.

**Figure 14:**
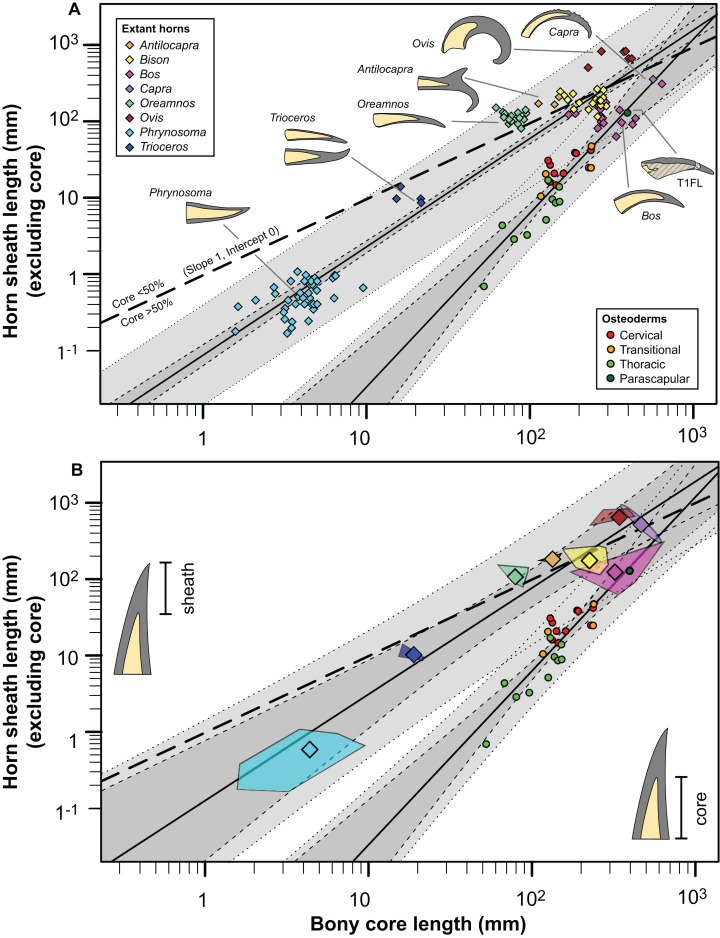
Comparison of horn core/sheath size and allometry in nodosaur osteoderms with modern cranial horns. Keratinous-only sheath length (i.e., the portion projecting beyond bony core) regressed as a function of bony core length, at both the specimen (A) level, and genus mean (B) level. Ordinary least squares regression indicated by solid line (Table 2), light dashed lines indicate 95% CI of the relationship, and dotted lines indicate 95% predication interval of points. Thick dashed line has a slope of 1 and intercept of 0. Diamonds indicate cranial horns, with small (A) representing individual horns, large (B) indicating genus means, and polygons (B) indicating minimum convex hulls. Circles indicate postcranial osteoderms of *Borealopelta*.

The rare studies documenting the size of both the bony and keratinous portions of horns in mammals (Borkovic, 2013; Bubenik, 1990; Grigson, 1975; Mead & Lawler, 1995), combined with similar squamate data obtained through either firsthand observations or digital measurements (Table S3) provide some initial context for both how these structures scale, and how they compare to the osteoderms of *Borealopelta*. A regression of the keratinous only portion of the horn (i.e., total length minus bony core length) regressed against the length of the bony core, shows a remarkably constrained pattern (*R*^2^ = 0.93), given both the disparate taxonomic sampling and more than three orders of magnitude in values on the data (Fig. 14). This regression is positively allometric (slope = 1.4) (Table 2), but uneven taxon sampling limits the statistical robustisity of the analysis. The smallest horned taxa sampled (i.e., the horned lizard *Phrynosoma*) show only minor contribution of the keratinous tissue to the overall horn length. Taxa with medium sized horns (i.e., the chameleon *Trioceros*) show nearly equal contribution of keratinous sheath and bony core to overall horn length. Cranial horns of mammals show variation in the relative contributions, but with many taxa showing approximately equal contribution of the keratinous and bony components. Within the sampled mammals, the largest relative keratinous sheaths are seen in those taxa with the largest horns (i.e., the caprids *Ovis*, *Capra*), whereas the smallest relative sheaths are seen in the domestic *Bos taurus,* and related *Bison bison* (both Bovinae).

**Figure 15:**
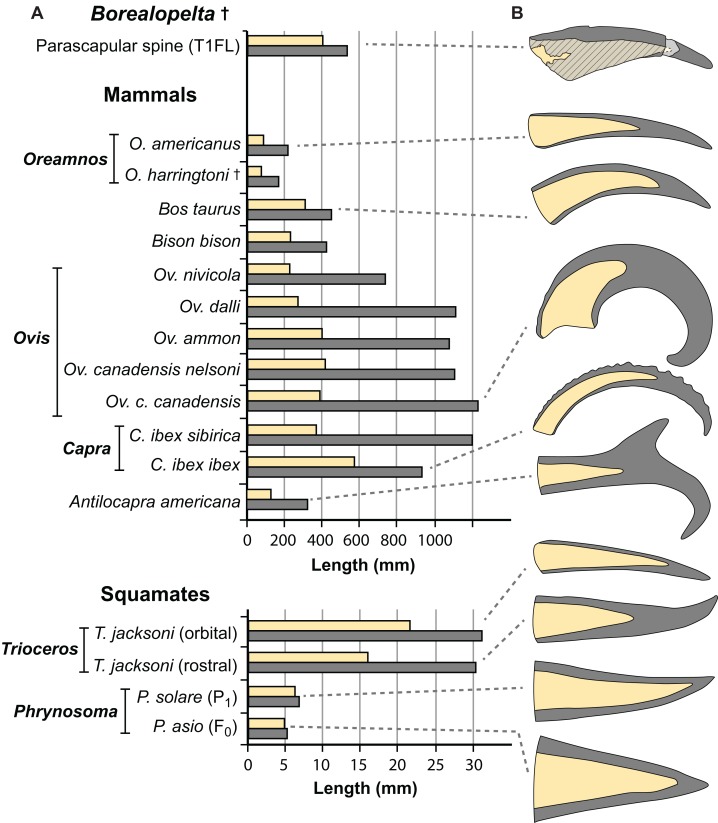
Comparisons of the size of the bony core and keratinous sheath of the parascapular spine of *Borealopelta* to modern bovid and squamate analogues. (A) Absolute size of the bone core (horncore or osteoderm) (yellow) and the overlying keratinous/horn sheath (grey) for the parascapular spine of TMP 2011.033.0001 (top) as well as averages for several bovid and squamate taxa (lower). (B) Schematic representations of the relative bony and keratinous components of select spines/horns (adjusted to same size). Data for *Oreamnos americanus* (*n* = 6, 20) and *Oreamnos harringtoni* (*n* = 10, 13) from Mead & Lawler (1995), *Bos* (*n* = 18) from Grigson (1975), *Antilocapra* (*n* = 3) and *Bison* (*n* = 18) from Borkovic (2013), *Ovis nivicola* (*n* = 2), *Ovis dalli* (*n* = 2), *Ovis ammon* (*n* = 2), *Ovis canadensis nelsoni* (*n* = 5), *Ovis canadensis canadensis* (*n* = 8), *Capra ibex sibirica* (*n* = 4) and *Capra ibex ibex* (*n* = 5) from Bubenik (1990), *Trioceros* (*n* = 1) from TMP 1990.007.0350, *Phrynosoma solare* (*n* = 1) from LACM 123351, and *P. asio* (*n* = 1) from WLH 1093.

Although the regressions of keratinous versus bony components of both *Borealopelta* osteoderms and extant squamate/mammal horns are positively allometric, the slope of the former (2.2) is greater than the later (1.4), resulting in convergence at large size (Fig. 14; Table 2). This means that for *Borealopelta*, although the sheaths of small osteoderms are quite modest, the larger the osteoderms the more similar their general morphology is to horns of extant analogues—specifically in terms of the relative size of the keratinous sheath. This is perhaps most clearly highlighted in the values for the parascapular spine (T1FL), which fall centrally within the spread of horn data from *B. taurus* (Figs. 11B, 14B and 15). This suggests that, at least in some cases, the cranial horns of Bovidae represent reasonable analogues in reconstituting both the relative and absolute horn/spine sheaths missing for the bony cores in ornithischians (Figs. 14 and 15).

Just how well the allometry of the spines of *Borealopelta* match those seen in display structures in a broader sample extant analogues, including additional mammals, squamates, and birds, is dependent on the collection of additional comparative extant data. Particularly limited are data for horned and casqued squamates (e.g., the chameleons *Trioceros* and *Chamaeleo*, the Sri Lankan horned agamid *Ceratophora*) and birds (e.g., hornbills—Bucerotidae, cassowarry—*Casuarius*). The best modern analogues remain the osteoderms of crocodilians, and additional data of the allometry and relative contribution of the bony osteoderms versus the keratinous scales would provide a better context in which to place the dermal armor of *Borealopelta* and other armored dinosaurs.

## Conclusion

The combined results showing that the osteoderm spines, and their keratinous coverings, are positively allometric (regionally); and that the anterior portion of the osteoderm series is both highly variable and has species specific morphology, provided new insights into the function and evolution of these structures. Similar results have been obtained from analysis of the exaggerated structures of most other ornithischian clades: Hadrosauridae (Dodson, 1975; Evans, 2010; McGarrity, Campione & Evans, 2013), Ceratopsia (Currie et al., 2016; Dodson, 1976; Hone, Wood & Knell, 2016; Lehman, 1990), Pachycephalosauria (Horner & Goodwin, 2009; Schott et al., 2011). These results in other ornithischian clades have been used to support the hypothesis that these exaggerated structures may have functioned, and evolved, in the context of socio-sexual selection (Hone, Wood & Knell, 2016; Hopson, 1975; Sampson, 1997). Similar hypotheses have been proposed for thyreophoran spines and plates (Carpenter, 1998; Hopson, 1977; Main et al., 2005; Padian & Horner, 2011), but until now had lacked commensurate morphometric backing. This argument is strengthened further when the parascapular spine is considered. Not only does this element show a different pattern of scaling than the rest of the series, but the absolute sizes of the keratin sheath and bony core are similar to the horns of extant bovids, and the relative sizes similar to the horns of some extant squamates, both of which are thought to function in socio-sexual display (Bustard, 1958; Farlow & Dodson, 1975; Geist, 1966). Combined with recent evidence suggesting this spine may, in life, have been pigmented differently than the rest of the osteoderms (Brown et al., 2017), this suggests this spine may have function as a visual socio-sexual display signal with conspecifics.

## Supplemental Information

10.7717/peerj.4066/supp-1 Supplemental Information 1 Linear measurements for the osteoderms of TMP 2011.033.0001. Linear measurements for the in situ osteoderms of TMP 2011.033.0001. See Fig. 4 for the osteoderm nomenclatural scheme.

10.7717/peerj.4066/supp-2 Supplemental Information 2 Linear measures for osteoderm length and keratinous sheath length in TMP 2011.033.0001. Linear measurements of osteoderms bony core length and keratinous only sheath length. See Fig. 4 for the osteoderm nomenclatural scheme.

10.7717/peerj.4066/supp-3 Supplemental Information 3 Dataset of extant mammal and squamate horn measurements for both the sheath and the core. Selection of horn measurements for extant mammals and squamates. Derived both from the literature and orignial measurements.

## Additional Information and Declarations

### Data Availability

The following information was supplied regarding data availability: The raw data has been provided as Supplemental Dataset Files.
